# Re-envisioning paradigms of education: towards awareness, alignment, and pluralism

**DOI:** 10.1007/s10459-021-10036-z

**Published:** 2021-03-19

**Authors:** Lindsay R. Baker, Shanon Phelan, Nicole N. Woods, Victoria A. Boyd, Paula Rowland, Stella L. Ng

**Affiliations:** 1 Department of Psychiatry, Faculty of Medicine, University of Toronto, Toronto, Canada; 2 Centre for Faculty Development, Faculty of Medicine, University of Toronto At St. Michael’s Hospital, 30 Bond Street, Toronto, ON M5B 2W8 Canada; 3 School of Occupational Therapy, Faculty of Health, Dalhousie University, Halifax, Canada; 4 Department of Family and Community Medicine, University of Toronto, Toronto, Canada; 5 The Wilson Centre, Unviersity of Toronto At University Health Network, Toronto, Canada; 6 Institute of Health Policy, Management and Evaluation, University of Toronto, Toronto, Canada; 7 Department of Occupational Science and Occupational Therapy, Faculty of Medicine, Toronto, Canada; 8 Department of Speech-Language Pathology, Faculty of Medicine, University of Toronto, Toronto, Canada

## Abstract

In this article we introduce a synthesis of education “paradigms,” adapted from a multi-disciplinary body of literature and tailored to health professions education (HPE). Each paradigm involves a particular perspective on the purpose of education, the nature of knowledge, what knowledge is valued and included in the curriculum, what it means to learn and how learning is assessed, and the roles of teachers and learners in the learning process. We aim to foster awareness of how these different paradigms look in practice and to illustrate the importance of alignment between teaching, learning and assessment practices with paradigmatic values and assumptions. Finally, we advocate for a pluralistic approach that purposefully and meaningfully integrates paradigms of education, enhancing our ability to drive quality in HPE.

Scholarship in health professions education draws from a range of disciplines and perspectives. Increasingly, these borrowed or imported scholarly methods and practices derive from the social sciences and humanities (Brosnan & Turner, 2009; Ousager & Johannessen, 2010), yet are often deployed without clear attention to their originating disciplines or “paradigms” of education. Kuhn defines a paradigm as a collection of beliefs shared by scientists, or a set of agreements about how problems are to be understood (Kuhn, 1962). Our use of the term paradigm, though inspired by, does not strictly adhere to, Kuhn’s definition. We use paradigms of education somewhat colloquially to mean a system of thought about educational principles and practices. Although principles and practices might be drawn from an array of disciplines outside of the health sciences, scholars from those external disciplines rarely publish their research in health professons education journals (Norman, 2011) nor do they necessarily participate in the translation of theory into health professions education (Martimianakis et al., 2009). Moreover, educators in the health professions often assume academic leadership roles without formal training in education theory or practice (MacDougall & Drummond, 2005; Srinivasan et al., 2011). This reality creates a paradoxical system in which those most responsible for the design and delivery of health professions education (HPE) have variable background in relevant content from education, psychology, sociology and the humanties. As a result, innovations in teaching and assessment can be launched and widely-adopted with questionable effectiveness and significant resistence due to misalignment with intended purpose or theoretical origins.

To begin addressing these concerns, we first present the background and history of different ways of thinking about education, drawn from the social science and humanities, and introduce a novel synthesis of education paradigms tailored to HPE. We then present two cases that demonstrate why paradigmatic awareness and thoughtful alignment between education paradigms and practices is advisable. And finally, we advocate for a pluralistic approach that purposefully and meaningfully integrates paradigms of education.

## Ways of thinking about education: an overview

Throughout history, scholars across disciplines have introduced a variety of ways to think about the underlying assumptions of education. For example, philosophers often focus on the overall purpose and goals of education, beliefs about what education institutions *should* teach, and the values and norms revealed through educational practice (Eisner, 1970; Noddings, 2018; Ornstein & Hunkins, 2017; Schiro, 2013). Historians are interested in how social, political and cultural influences have shaped what is valued and included in the curriculum throughout various points in history (Kliebard, 2004; Ornstein & Hunkins, 2017). Psychologists organize around shifts in knowledge bases in the study of human and child development (McInerney, 2013; Ornstein & Hunkins, 2017). And sociologists analyze education according to its role in people’s lives, the social/economic foundations on which it is based and the public goals schools *should* aim to achieve (Ballantine & Hammack, 2009; Clabaugh & Rozycki, 1990; Ornstein & Hunkins, 2017). Others discuss education based on how underlying assumptions and beliefs are enacted on a practice level related to curriculum, teaching and learning. For example, curriculum theorists describe different conceptualizations or ways of thinking about what makes up curriculum (Smith, 2000), education psychologists group different learning theories together based on similar approaches to how people learn (McInerney, 2013; Ornstein & Hunkins, 2017), and education theorists categorize according to different teaching approaches or perspectives (Miller & Seller, 1990; Pratt, 2002). These different ways of categorizing education, while we have only scratched the surface, illustrate the diversity and complexity of ways of talking about and thinking about the purpose, practice and value of education. Assumptions such as these become implicit in education scholarship and practice. Because the original theorists tend to remain working in their own disciplines, the underlying assumptions of borrowed ways of thinking and practicing can be taken for granted after uptake into another field.

In HPE, the language of psychological perspectives and theories are commonly drawn upon to frame curricular decisions and day-to-day educational practices. Yet psychological perspectives do not fully represent the spectrum of educational approaches at work in HPE. Arguably, when a field gives primacy to any one discipline’s framing it can be helpful to step back to consider other philosophical assumptions, epistemological origins and historical, political and sociocultural influences associated with these perspectives and theories. Taking this step can help broaden perspectives and check assumptions, lest they constrain the progression of knowledge. As a first step in this direction, we synthesize educational, psychological, philosophical and sociological perspectives into a framing of *paradigms of education for the health professions.* To do this, we looked for commonalities in the multiple ways of thinking about education across disciplines and grouped accordingly, using language common to HPE for relatability. The purpose of this framing is to integrate diverse ideas from other disciplines and capture education work that sits outside the margins of dominant approaches in HPE (Baker et al., 2019) to propose a pluralistic approach to thinking about education.

## Paradigms of education for the health professions: a novel synthesis

We introduce six major paradigms of education that are relevant to HPE. These paradigms represent our synthesis of many of the educational ideologies and categorizations above. For each, we link a philosophy with origins in psychology with a synthesized description of what that philosophy could create (Ballantine & Hammack, 2009; Clabaugh & Rozycki, 1990; Eisner, 1970; Kliebard, 2004; Miller & Seller, 1990; Ornstein & Hunkins, 2017; Pratt, 2002; Schiro, 2013; Schuh & Barab, 2007; Smith, 2000)**.** The paradigms overlap, but differ meaningfully with respect to their philosophical assumptions about what exists in the world (ontology) and how we come to know about what exists (epistemology). Each paradigm has a nuanced perspective on the purpose of education, the nature of knowledge, what knowledge is valued and included in the curriculum, what it means to learn and how learning is assessed, the roles and nature of teachers and students in the learning process. We aim to clarify these distinctions and the underlying assumptions associated with each paradigm. We also identify the limits of each paradigm and when and where other paradigms may offer complementary perspectives and practices. For the purposes of this paper we are focusing on formal education and curriculum, recognizing that this is but one part of the education enterprise. While we present each paradigm as a separate entity in a sequence, there is also temporal overlap and each subsequent paradigm does not render the previous invalid. Our naming convention of "philosophy-goal" means the first word describes the philosophy, and the second word describes our interpretation of aligned goals.

### Behaviourism-itizenship

The overall purpose of education in the Behaviourism-Citizenship paradigm is to shape desirable behaviours toward preparing learners to behave as valuable members of society. Within this paradigm there is one external reality and knowledge of singular truths are able to be acquired by individuals. Learners are considered blank slates, and learning involves acquiring correct information about the world. Learning or knowledge acquisition is viewed as a causal process, just like any other natural phenomenon, and the desired outcome of learning is a change in form or frequency of observable behaviour (what people say or do) (Watson, 1913). Teachers, who have mastery of subject matter, shape this observable behaviour by transmitting knowledge to learners, using systematic conditioning and reinforcement (McSweeney & Murphy, 2014; Pavlov & Anrep, 2003). Reinforcement increases the likelihood that a specific behaviour will occur more frequently in the future by delivering or removing a stimulus immediately after a behaviour. Desired behaviours thus become habits, traits or dispositions as they are reinforced and honed by use over time. Assessment and evaluation focus on learners demonstrating these desired behaviours, measured against external standards (for example, entrustable professional activities) (Ten Cate et al., 2015). There are many instances in health professions where a behaviourist approach to inscribing proper performance may be most effective. For example, in managing infection prevention and control and donning / doffing personal protective equipment, we need and want clear protocol-driven, heavily regulated procedures. The creation of a citizen identity means that the performance of this process in a rote and standard manner is necessary to the greater good. The Behaviorist-Citizenship paradigm faces critiques of becoming a form of control, regulation, and surveillance which is counter to the ideals of education and learning as avenues for societal advancement and forms of personal growth (Hodges, 2015).

### Cognitivism-Expertise

The Cognitivism-Expertise paradigm extends the behaviourist paradigm beyond knowing what to do, toward understanding why and when the behaviours may or may not be appropriate. This shift thus develops experts who can respond more flexibly, as opposed to ‘good citizens’ who can conform. Knowledge is still framed as content or information external to the learner, however the focus here is on how this information is stored in memory. Rather than focusing on observable behaviours, the cognitivism-expertise paradigm is concerned with development of the unobservable mental structures and processes within the mind, which define what learners know and frames how they come to acquire new knowledge. Learning is considered by definition to involve the acquisition of knowledge through senses, experiences or formal instruction by teachers. Learners are viewed as information processors and teachers facilitate this processing through focused attention on how information is structured, organized and retrieved, and transferred to new situations. For example, studies of integrated instruction have demonstrated that teaching the conceptual knowledge associated with clinical or procedural knowledge results in more accurate/expert practice (Bandiera et al., 2018; Mylopoulos et al., 2017). Assessment and evaluation within this paradigm focus on retention and near transfer of learning (when many elements overlap between the conditions in which the learner obtained the knowledge or skill and the new situation) (Castillo et al., 2018). The cognitivist paradigm faces critiques for being too narrowly focused on mental representations in the mind, at the expense of how knowledge is socially constructed, shared, and negotiated.

### Constructivism-Expertise

The Constructivism-Expertise marks an ontological and epistemological shift about the nature of knowledge. Knowledge isn’t something external to learners, which they acquire; knowledge is something learners actively “construct” as they make meaning of their experiences (Piaget, 1953). Within this paradigm, the focus is on understanding mental representations to support learners in constructing new knowledge. Knowledge is seen as dynamic, with new knowledge being constructed by learners upon prior knowledge, in order to solve novel problems (Dewey, 1938). The goal of education in this paradigm remains creating experts, and adaptive expertise exemplifies the dynamic nature of knowledge required to enact expert practice (Mylopoulos & Regehr, 2011; Mylopoulos et al., 2018). Learners engage in activities designed to promote discovery of knowledge and the teacher’s role is to provide appropriate resources and support for each stage of cognitive development. A constructivist curriculum focuses less on specific content, and more on the process of knowledge construction. Learners’ previous knowledge is a key consideration in effective teaching and curricular planning. Assessment focuses on application or transfer of knowledge to novel contexts and preparation for future learning (Mylopoulos et al., 2016; Schwartz & Martin, 2004). This paradigm emphasizes the learner and formal education, as opposed to learners’ broader interactions in society.

### Constructivism-Interlocution

The turn toward Constructivism-Interlocution begins the social focus of education. Constructivism-Interlocution posits that the creation of knowledge cannot be separated from the social environment in which it is formed. Whereas constructivsm-expertise focuses on understanding mental representations, constructivsm-interlocution is focused on the ways in which knowledge is constructed through sociocultural influences and interaction (Vygotsky, 1980). Learning is understood as identity formation and the co-creation of knowledge. This is thought to happen through participation in social contexts and *enculturation*—picking up the jargon, behaviour, and norms of a new social group, and adopting its belief systems to become a member of the culture (Brown et al., 1989). Indeed, the function of education is seen as a means to socialize learners to be active participants in communities. Learners are active participants and the role of the teacher is to facilitate social interactions and collaborative work. The constructivism-interlocution paradigm accounts for workplace-based learning and assessment, and brings forward more informal approaches to learning like communities of practice (Lave, 2004; Wenger, 1998). However, eductional goals are still constructed and structured within formal institutions according to established systemic expectations. In the following paradigms (humanist-self-acualization and transformative-change agency), learning for oneself takes more of a focus as opposed to learning for a particular mandate, and systems and structures themselves are challenged.

### Humanism-self-actualization

The humanism-self-actualization paradigm aims to prepare learners to progress towards the realization or fulfillment of one's full potential and autonomy (self-actualization) (Maslow, 1943). This paradigm is focused on engaging the learner as a whole, including both cognitive and affective domains. The goals of education take into consideration the learner as a person, attending to learning in relation to emotional and physical wellbeing. To achieve these goals, schooling is learner-driven and learning is viewed as the achievement of one’s personal goals. Learners’ choice and control over education are emphasized, with teachers facilitating and nurturing the learning process. This paradigm views the function of the curriculum as providing personally satisfying experiences for each individual learner. As such, self-evaluation is the only meaningful assessment within this paradigm—external grading is viewed as irrelevant and is thought to encourage students to work for a grade and not for personal satisfaction. The humanist-self-actualization paradigm arguably focuses too much on the individual, a critique that is compounded by evidence that generally, individuals are not able to accurately self-assess (Eva & Regehr, 2008).That said, when channeling self-assessment in relation to self-actualization, the goal of self-assessment may not be to determine the level and accuracy of knowledge gains. Rather, it may be to determine whether an individual feels satisfied with their journey toward their own pre-determined goals. This shift in object of self-asessment complicates the mantra that we are poor at self-assessment. Indeed personal meaning and motivation are overlooked aspects of learning and humanist paradigms may offer an important angle into promoting engagement in education (Kusurkar & Croiset, 2015). Given these paradigmatic points, the reflective portfolios so commonly used in medical education to document goals and journeys toward them would likely best be situated within the humanism-self-actualization paradigm of education (Driessen et al., 2007).

### Transformation-change agency

Finally, the aim of the transformation-change agency paradigm is to empower learners to see the social world through a continually more ethical lens, so that they will challenge and change the status quo as agents of change toward a more just society (Freire, 1993; Hooks, 1994). This paradigm views knowledge as a social construction. Learning is viewed as a shift in perspective toward a critically reflective way of being, which continually challenges individual and societal assumptions and practices, with attention to ethics, justice, and power. Traditional approaches to education are seen to reinforce the status quo, perpetuating inequalities in society and contributing to the oppression of learners themselves (Freire, 1993; Hooks, 1994). Within the transformation-change agency paradigm, the learner-teacher distinction is minimized. Social reform and responsibility to the future of society are central, beginning by raising the learners’ own awareness of dominant power relations and structures. Assessment focuses on shifts in perspective—conversation foci/content during debriefs and dialogue or responses to systems level scenarios. The transformative-change agency paradigm has faced critique for glossing over requisite knowledge and skill. Additionally, to be transformative is inherently counter-culture; thus transformative education presumes agency that some learners may genuinely lack. At the same time, the advantage of the transformative-change agency paradigm is that it targets education beyond the individual. In instances where existing educational approaches have not fully realized their goals, transformative approaches may offer a way forward. For example, we have seen that interprofessional education alone cannot address some of the hierarchical, workplace-based aspects of interprofessional practice (Baker et al. 2011; Paradis & Whitehead, 2015). Transformative and critical education efforts may be warranted to help different professionals understand the socially-situated nature of knowledge work and how systems can drive competition between professions. This understanding can then underpin efforts to disrupt unhelpful hierarchies and move toward genuine collaboration. Dialogic education represents one transformative education approach to addressing power relations in a safe and productive manner (Bakhtin, 1981; Kumagai & Naidu, 2015).

These brief syntheses serve as introductions to what we argue is prerequisite knowledge for applying educational approaches from multiple disciplines. Paradigmatic misalignment or incongruence can have discursive and material consequences; we offer three such examples of misalignment common in HPE. Consider the many critiques of reflection. Some have argued it has become a mechanism for surveillance and thus an inauthentic, burdensome demonstration, instead of a meaningful approach for learning (Hodges, 2015; Nelson & Purkis, 2004). Others have expanded on this argument to suggest that the pressure to assess reflection according to dominant ways of thinking about learning and assessment are to blame for these instrumental uses of an educational concept that was initially an attempt to balance the dominant, technical-rational approaches to professional practice (Ng et al., 2015). This example demonstrates unintended negative consequences when an educational approach, deriving from a humanism-self actualization or transformation-change agency paradigm, is applied without paradigmatic awareness in contexts that are dominated by practices incongruent with these paradigms (e.g. rubric-based assessment). In a recent study examining learners’ epistemological beliefs in relation to simulation-based education, Ng et al. (2019) identified incongruence between the widely held beliefs about simulation as a safe space for learning and learners’ sense that they needed to perform with certainty and confidence at all times. This example shows that the epistemological culture or climate also impact the ability to align paradigms with practices. And finally, consider the attempts to apply social sciences and humanities approaches to HPE. A focus upon adding a sufficient “dose” of humanities content into curricula rather than the actual purposes of health humanities can limit meaningful application (Bishop, 2008; Tsevat et al., 2015) as well as “overstuff” the curriculum without commensurate benefit (Whitehead & Kuper, 2012). Focusing on dose while failing to attend to humility, power, and learner safety can actually make the teaching of humanities unhelpful within health professions programs. A humanism-self-actualization or transformation-change agency paradigm may be required to fully realize the goals of these intiatives. Attention to paradigms can prevent simply adding another content area while potential benefits of the content are lost.

## Toward paradigmatic awareness and alignment

To foster awareness of how different paradigms look in practice and to illustrate the importance of alignment between the teaching, learning and assessment practices with paradigmatic values and assumptions, we present two case examples. We will walk through what an instance of ethics education in the health professions might look like, when informed by two different education paradigms (see Table 1). Neither example is meant to be positioned as better or worse than the other. Rather, they are meant to highlight how different paradigms might approach teaching for the same ultimate goal: ethical practice. The cases illustrate that the same goal—preparing practitioners to be able to practice ethically—takes a different starting frame depending on paradigm: teaching reasoning that supports ethical decision-making versus inspiring a virtuous orientation that underlies and supports ethical practice. These frames set the stage for how teaching and assessment unfold, as well as for what exactly is considered a part of teaching and assessment. Notice the greater emphasis on testing in the cognitivism-expertise example and the greater emphasis on tone-setting for the transformation-change agency example. This is not to say a cognitivism-expertise educator would not set the tone or a transformation-change agency educator would not assess learning; however, outcomes and how they are prioritized look different for those more strongly influenced by one paradigm over another. Awareness of these influences matters because a lack thereof could result in uninformed and unproductive paradigmatic misalignment. For example, imagine the incongruence that would result if an instructor used cognitivist testing approaches after asking learners to share personal stories of ethical tensions from practice! Our two case examples demonstrate how focused attention on alignment can help strengthen the connection from educational design to educational outcomes.

**Table 1:** Case examples of ethics education from different education paradigms

*Case 1: Cognitivism-Expertise Paradigm*
An educator most strongly influenced by the cognitivism-expertise paradigm of education likely begins by considering what it means to prepare practitioners who can engage in ethical reasoning and practice. They aim to identify the appropriate knowledge required to support this definition of ethical practice. This body of knowledge includes knowledge of ethics and its associated principles. So, this educator aims to provide students with relevant experience and knowledge of concepts and approaches to ethical tensions. The educator looks to prepare learners for desirable outcomes (how the students identify and act on a particular ethical tension) and processes (how they arrive at their decisions or course of action). The educator also endeavours to understand whether the learning can transfer to novel cases with both similar and dissimilar contexts in the future.
An informed lesson aligned with a cognitivism-expertise orientation involves splitting students into groups of 5–7 to discuss cases in which a variety of ethical tensions arise. Learners read each case thoroughly, and then as a group identify ethical tensions, think about the reasons why they are ethical tensions, and brainstorm how to resolve them. The educator carefully designs/selects these cases to introduce learners to ethical tensions not yet discussed in class. The purpose of this activity is not for learners to get the correct answer, but rather to provide experience working with ideas and approaches that the educator will elaborate upon after the activity. Following the activity, the educator provides direct instruction about different types of ethical tensions and various strategies for addressing them. The educator also highlights underlying commonalities and differences across the cases and provides learners with a chance to ask questions for clarification. To initially assess learning, learners identify ethical tensions in novel cases with similar contextual variables (e.g. similar patient population or team makeup), discuss their reasoning and describe how they would respond in each case. A final assessment tests their ability to transfer their learning when contextual variables are changed. Ultimately, the final assessment determines if learners have the ability to practice ethically, per the initially identified definition of ethical practice. The learners’ ability to do so is built upon a foundational knowledge base explored in class.
*Case 2: Transformation-Change Agency Paradigm*
An educator most strongly influenced by the transformation-change agency paradigm of education learning likely sees ethical practice as virtuous practice. That is, rather than thinking and speaking about ethical reasoning based on a set of agreed upon knowledge and deliberation methods, this educator thinks and speaks about ethical ways of being and seeing. In this orientation, the educator is concerned with what a learner would want to do and what they would do even if no one were assessing or watching at all, rather than prioritizing demonstrable application of knowledge to novel situations. Thus the goal of ethics education here take the shape of creating a safe, reflective, theory-informed space for learners’ consideration of ethical tensions and ethical action.
This orientation toward virtuous practice may be novel to a student accustomed to a system of memorizing, understanding, applying, building, and demonstrating knowledge in a particular way. This approach begins by re-setting the tone for learners in terms of their education expectations, in order to orient them to transformative pedagogy. The type of learning to ensue requires a degree of vulnerability, and management of ambiguity and uncertainty, which learners may not be accustomed to in their education to date. Where other courses have offered an evidence-based practice approach to determining the best treatment plan for a patient, this particular session emphasizes the consideration of patient, professional, and societal values. Thus prior to class, the educator prepares students by explaining that the next class may feel a bit different, that there will be some sharing about challenging topics, and that students will not be judged for their perspectives but rather for their willingness to openly listen to one another and challenge their own assumptions.
To further help set the tone, learners are assigned an article about ethical tensions experienced by health professions students and are asked to start thinking about an ethical tension they have experienced personally. In groups of 5–7, learners engage in dialogue about the tensions they have identified, with the following guiding questions: Reflect and discuss your initial reactions, How did you discern it was in fact an ethical tension?; Identify the types of ethical tension(s) involved; What or who might you draw on to guide your deliberation and decision making?; What actions might be taken to seek resolution?; If applicable, what did you do and would you do anything differently?
Bringing it back to the large group the educator then shares a personal story of an ethical tension she experienced in practice. She chooses to do this to demonstrate her own vulnerability as the “teacher,” which disrupts the traditional teacher-learner hierarchy and supports learner safety within the learning experience. As a group the class then engages in a dialogue about ethical tensions in practice. For the most part this section is conversational, although there are parts that are more didactic where terminology and constructs relating to ethical practice and ethical theory are explored.
At the end of the class, the instructor provides resources for self-care and offers follow-up availability to support students who may need to further process, discuss, and debrief about their reflections and experiences.

Beyond the classroom context, at the broader institutional level, the tensions between paradigms become apparent when looking at how the purpose of higher education has been historically understood. For example, up until the twentieth century, few people attended college. With their financial futures secure, students sought to receive a liberal arts education in order to “refine their comprehension of the virtues of civic participation in a society that they would one day come to shape” (Raelin, 2007, p. 58). It was not until the middle of the twentieth century that higher education became associated with professional education. This association was a significant shift. No longer just about civic participation for the elite, colleges and universities were in the business of creating employable graduates. In the presence of increased competition for attracting students, these colleges and universities attempted to establish their reputation and standing by continuing to implement strategies that had animated their success as liberal arts colleges. These strategies included hiring prestigious academics, encouraging a strong emphasis on research, and developing disciplinary knowledge. These strategies—while successful for liberal arts colleges and universities—became too far removed from the real world of practice and had the paradoxical effect of devaluing the educational institutions and their possible contribution. This historical tension about the purpose of higher education demonstrates why paradigmatic awareness matters at the institutional level. Belief systems about whether the purpose of higher education is about preparing citizens or creating employable graduates—while not mutually exclusive rationales—do shape higher education institutions. Raelin (2007) proposes the steady shift away from liberal arts towards professional education, with its associated emphasis on skills and competencies, has been one of the most underrecognized trends in higher education in North America. Attending to these trends on an institutional and societal level has led some concerned sociologists to question: if our education systems continue to tilt away from the liberal arts, will our societies have the social and political literacy that we need to live with the technological advances we are creating (Benjamin, 2013)? We add to this question: what are the implications of unchecked paradigmatic assumptions in an increasingly global health professions education context? (Martimianakis & Hafferty, 2013). Given the socio-cultural and socio-political influences on education, how might unearthing paradigms of education impact international collaborations and knowledge mobilization efforts? All this to say, the tensions between paradigms that show up in the everyday practices of education also manifest in shaping the institutions of education and thus awareness of (mis)alignment is prudent for all educators.

## Toward a pluralistic approach

As our synthesis of paradigms demonstrates, many different interests guide education, and HPE initiatives operate in complex contexts, embedded within academic hospitals, community settings, and large higher education organizations (universities and colleges). This complexity has led to a sense of competing demands on learners, and potentially influenced the effects of the education initiatives themselves: even the best laid education plans are subject to “countervailing forces” upon them (Rowland et al. 2019). Indeed we are not arguing for paradigmatic purity. Instead we are calling for a *paradigmatic pluralism,* i.e. an approach to education characterized by paradigmatic awareness, alignment and—when appropriate, purposeful misalignment. Foundational to this approach is the understanding that educational goals and foci may differ between stakeholders, expand, or change; and thus new outcomes of interest must be attended to and assessed, without discarding prior effective approaches and theories. And understanding of and attention to paradigms of education is a prerequisite to pluralism, as we would suggest one cannot optimally mix what one does not deeply understand.

Pluralism is even more important given the consideration of paradigms needs to happen at all levels of the curriculum and across the full spectrum of education. Curricula operate at the level of program, course and session (Goldman & Schroth, 2012). And while a health professions training program may operate primarily within one or two paradigms of education, other paradigms may be present at the course or session level where they may flounder or flourish. Further, organizations impose their own priorities and constraints upon curriculum developers, teachers, and students. Thus if we *do* consider all paradigms of education, then the bounds of what education entails also expands. It includes not only curriculum and pedagogy; it also includes the broad educational values that confront a student from the moment they review a website to consider applying to a program (Razack et al., 2015) and the way that admissions committees decide upon which students to admit to their programs (Wright, 2015).

While consideration of paradigms, their internal alignment, and their meaningful mixing are what we propose, we also acknowledge that purposefully applying a range of practices from multiple paradigms of education will not come without challenges. The consideration of multiple paradigms also requires attention to the notion of evidence-based and theory-informed education (Fenwick, 2016; Greenhalgh et al., 2003; Trisha Greenhalgh, 2010; Horsley & Regehr, 2018; Regehr, 2010; Van Der Vleuten et al., 2000). When drawing from data and theories derived or generated from different epistemological traditions, multiple definitions exist for what is “best,” what is high quality, what is valid. Biomedical and psychosocial approaches dominate medicine; cognitivist- and constructivist approaches are thus most amenable to HPE. These approaches fit well together because they have similar epistemologies. All draw upon a dominantly agreed upon scientific method and the conceptions of knowledge this method can produce. Humanistic approaches differ, epistemologically, in that their concern is far more personal and thus outcomes are more challenging to demonstrate according to prevailing evidence-based approaches. Transformative approaches inherently challenge dominant approaches—not in terms of their scientific claims, but in terms of the sociopolitical effects of these claims, by asking questions like who benefits from this particular framing of ethics, or professionalism, or disability/disease? Who is harmed by it?

Indeed paradigms of education that position the teacher and learner more as partners in knowledge creation or discovery thus also challenge traditional hierarchies. To be transformative as an educator is to challenge the status quo. That said, we do not propose that one must completely overthrow established health professions education systems. We believe that each paradigm has merits for certain aspects of health professions education. For example, when teaching infection control, a behaviourism-citizenship paradigm, though the most dated of all the paradigms, may offer useful practices. Yet bridging it with other paradigms could prove more helpful, hence our argument for pluralism. But pluralism requires attention to the paradigms and practices outlined in this paper as well as potentially thoughtful consideration of complex and network based theoretical framings. Future conceptual work on this topic could explore how theories like actor-network theory, activity theory, and others (Fenwick, 2016) could contribute to pluralistic approaches to education. This discrepancy between the dominant paradigms of HPE and the humanism-self-actualization and transformation-change agency paradigms creates a tension in integrating these approaches into HPE (Kuper et al., 2017). To move past or work productively with such tension requires a critical questioning of each educator’s own assumptions about education. And at the systems level, it requires a careful look at the broader systems driving education in society, workplaces, and health professions training programs.

## Conclusion

A pluralistic approach to education would see educators knowledgeable about multiple paradigms of education, and adept at paradigmatic alignment. Educators would be able to carefully consider how the different paradigms work together and where they conflict, so they can help ensure clear, aligned educational practices that are evaluated appropriately. This alignment matters particularly during a time of widespread curricular reform efforts within health professions training programs, wherein performance-driven outcome measurement rules academic health science systems. If educators lack awareness and fail to align, they may not only promote sub-optimal education, but may also be unable to appropriately evaluate educational efforts or demonstrate meaningful impacts where they exist. With alignment, the inevitable pluralism of paradigms of education can be engaged well, increasing our ability to drive quality in health professions education. Informed pluralism could offer a hedge against slipping into dogmatic or unthoughtful approaches to education in the health professions.
